# Intra-generational social mobility and mortality among older men in the Concord Health and Ageing in Men Project: A cohort study

**DOI:** 10.1016/j.ssmph.2023.101581

**Published:** 2023-12-11

**Authors:** Minh-Hoang Tran, Anita van Zwieten, Kim M. Kiely, Fiona M. Blyth, Vasi Naganathan, David G. Le Couteur, David J. Handelsman, Markus J. Seibel, Louise M. Waite, Robert G. Cumming, Saman Khalatbari-Soltani

**Affiliations:** a School of Public Health, Faculty of Medicine and Health, University of Sydney, Sydney, NSW, Australia; b NTT Hi-Tech Institute, Nguyen Tat Thanh University, HCMC, Viet Nam; c Centre for Kidney Research, Children's Hospital at Westmead, Westmead, NSW, Australia; d Ageing Futures Institute, University of New South Wales (UNSW), Sydney, (NSW), Australia; e ARC Centre of Excellence in Population Ageing Research (CEPAR), University of Sydney, Sydney, NSW, Australia; f Concord Clinical School, Faculty of Medicine and Health, University of Sydney, NSW, Australia; g Centre for Education and Research on Ageing, Faculty of Medicine and Health, University of Sydney, New South Wales, Australia; h Ageing and Alzheimer's Institute, Concord Repatriation and General Hospital, Sydney Local Health District, Concord, New South Wales, Australia; i ANZAC Research Institute, University of Sydney and Concord Hospital, Sydney, Australia; j School of Health and Society and School of Mathematics and Applied Statistics, University of Wollongong, Wollongong, (NSW), Australia

**Keywords:** Intra-generational, Social mobility, Mortality, Older adults, Socioeconomic position, Life-course

## Abstract

**Objectives:**

We examined associations between intra-generational social mobility (reflected in life-course socioeconomic trajectories) and mortality, among older men.

**Methods:**

Data came from a prospective Australian community-based cohort of older men. Social mobility was defined by socioeconomic indicators from three points in the life-course: educational attainment (late adolescence-early adulthood), occupation (mid-life), and current sources of income (older age). We defined indicators of social mobility trajectory (6 categories; reflecting the direction of social mobility) and social mobility status (2 categories; mobile or non-mobile). We used Cox regression to examine associations with mortality, adjusting for age, country of birth, and living arrangement.

**Results:**

We followed 1568 men (mean age 76.8, SD 5.4) for a mean duration of 9.1 years, with 797 deaths recorded. Moving upward was the predominant social mobility trajectory (36.0%), followed by mixed trajectories (25.1%), downward (15.1%), stable low (12.2%), stable high (7.6%), and stable middle (4.0%). Men with downward (Hazard ratio 1.58, 95% CI 1.13 to 2.19) and stable low socioeconomic trajectories (1.77, 1.25 to 2.50) had higher mortality risks than men with stable high socioeconomic trajectories, while men with upward trajectories had similar risks to those with stable high trajectories. 76.2% of the participants were classified as having mobile status; no associations were evident between binary social mobility status and mortality.

**Discussions:**

These findings suggest cumulative and persistent exposure to disadvantaged socioeconomic conditions across the life-course, rather than social mobility, is associated with increased mortality. For each stage of the life-course, addressing socioeconomic disadvantage may reduce inequities in mortality.

## Highlights

• Evidence is scarce on intra-generational social mobility and mortality among older adults.
• We used three different socioeconomic indicators in three distinct life-course periods to evaluate associations between intra-generational social mobility and mortality, using a combination of theory-based hypothesis approaches and a data-driven approach.
• Our findings suggest cumulative and persistent exposure to disadvantaged socioeconomic conditions across the life-course, rather than social mobility (e.g., downward mobility), is associated with increased mortality.

## 1 Introduction

An extensive body of evidence documents the impacts of socioeconomic conditions experienced throughout the life-course on later-life health outcomes (Kuh & Ben-Shlomo, 2004), including the association of poorer socioeconomic conditions with shorter life expectancy (Gallo et al., 2012; Mackenbach et al., 2008; Marmot, 2005; Tawiah, Jagger, Anstey, & Kiely, 2022). However, individuals’ socioeconomic conditions are not necessarily stable over time and it is less clear how changes in socioeconomic conditions across the life-course influence mortality. A number of theoretical models have been proposed to explain how life-course socioeconomic conditions might be connected with health and mortality outcomes in older age (Kuh & Ben-Shlomo, 2004). Accumulation models propose cumulative, dose-response relationships between adverse socioeconomic experiences over time and later outcomes. Acculturation models suggest that the effects of destination socioeconomic conditions on health outcomes are stronger than the effects of socioeconomic conditions of origin (Schumann et al., 2020). Critical period models hypothesise that adverse early-life socioeconomic circumstances have independent, harmful effects on health (Kuh & Ben-Shlomo, 2004; Lynch & Smith, 2005). Social mobility models, the focus of this study, place greater emphasis on changes in individual socioeconomic conditions over time (Kuh & Ben-Shlomo, 2004). These mobility trajectories can be downward, upward, stable, or may involve intermittent fluctuations.

There are two prominent social mobility hypotheses with contrasting views 1) trajectory based explanations, including so-called ‘falling from grace’ and ‘rising from rags’ hypotheses (Gugushvili, Zhao, & Bukodi, 2019) and 2) non-directional explanations—the dissociative hypothesis. Falling from grace and rising from rags hypotheses suggest that downward or upward life-course movement in social position is associated with poorer or improved health outcomes, respectively. The dissociative hypothesis suggests that moving among social classes in any direction is likely to be associated with poorer health outcomes, above and beyond influences of one's prior or current socioeconomic conditions, due to psychological distress arising from conflicts between former and current socioeconomic conditions (Sorokin, 1927). To date, most studies examining the links between life-course social mobility and health have focused on inter-generational dynamics whereby change in social position is considered across generations; these associations have been examined in relation to a range of outcomes including self-rated health, well-being, lung function, and cognitive function (Assari, 2018; Gugushvili et al., 2019; Jonsson, Sebastian, Hammarström, & Gustafsson, 2017; McLoughlin, Präg, Bartley, Kenny, & McCrory, 2023; Stringhini et al., 2018; Tiikkaja & Hemstrom, 2008; Veenstra & Vanzella-Yang, 2021; Zang & Kim, 2021). This body of research has shown increased risks of adverse health outcomes in some mobile groups, particularly those with downward inter-generational social mobility (Assari, 2018; Gugushvili et al., 2019; Stringhini et al., 2018; Tiikkaja & Hemstrom, 2008; Veenstra & Vanzella-Yang, 2021; Zang & Kim, 2021).

Few studies have examined the relation between intra-generational social mobility and mortality where change in social position is considered within households or individuals (Billingsley, 2012, 2019, 2020; Claussen, Smits, Naess, & Davey Smith, 2005; Ericsson, Pedersen, Johansson, Fors, & Dahl Aslan, 2019; Hart, Smith, & Blane, 1998; Nguyen & Nguyen, 2020; Pensola & Martikainen, 2003; Uggla & Billingsley, 2018). Most of these studies have been conducted in Europe or the United States and have evaluated social mobility based on a single indicator of socioeconomic conditions over time, mainly occupational position (Billingsley, 2012, 2019, 2020; Hart et al., 1998; Uggla & Billingsley, 2018). Several studies have investigated the risk of mortality across only two stages of the life-course (Claussen et al., 2005; Hart et al., 1998; Pensola & Martikainen, 2003), limiting the trajectories of intra-generational social mobility that could be examined. Many have focused on young to middle-aged people (Billingsley, 2012, 2019, 2020; Claussen et al., 2005; Hart et al., 1998; Nguyen & Nguyen, 2020; Pensola & Martikainen, 2003; Uggla & Billingsley, 2018), meaning the impact of social mobility on mortality at older ages, when the burden of mortality is at its greatest, remains unclear. These studies mainly found that individuals with downward social mobility had higher mortality than those who were consistently advantaged, but the downwardly mobile group did not necessarily have higher mortality than those who were always disadvantaged (Ericsson et al., 2019; Hossin, Heshmati, Koupil, Goodman, & Mishra, 2022). Evidence on intra-generational social mobility and mortality among older adults is scarce; findings from these studies were mainly similar to those that included wider age ranges, with the highest mortality reported among those with downward social mobility (Ericsson et al., 2019; Nagamine et al., 2020) as well as those with life-time low socioeconomic position (Ericsson et al., 2019). No study in Australia has so far examined such associations among older adults. Of note, the opportunities that men and women experience during their life-course differ systematically, indicating the need for gender-specific analyses. While some studies reported gender differences in intra-generational social mobility and all-cause mortality among different age groups and contexts (Billingsley, 2019; Nagamine et al., 2020), these differences are not consistently found in available literature (Ericsson et al., 2019; Hossin et al., 2022). Given the highly gendered segregated nature of the labour market, particularly before the 1970s, women in older generations had distinct labour market experiences, and traditionally, their socioeconomic position was tied to their partner's position (Næss, Hernes, & Blane, 2006; Tiikkaja, Hemström, & Vågerö, 2009). Thus, due to lack of data and difficulties in accurately assessing the social mobility of older generations of women in the same way as for men, this study focuses on older men.

Given limited evidence on intra-generational social mobility and mortality among older adults, in this study we investigate the association between intra-generational social mobility and mortality among older men, focusing on the falling from grace/rising from rags and dissociative hypotheses as well as data-driven approaches (Hossin et al., 2022).

## 2 Methods

This study followed the Strengthening the Reporting of Observational Studies in Epidemiology (STROBE) reporting guideline (Supplementary Checklist 1).

### 2.1 Study population

We used data from the Concord Health and Ageing in Men Project (CHAMP), a long-term population-based cohort study (Cumming et al., 2009). Men aged 70 years or older who resided in a defined geographical region (the local government areas of Burwood, Canada Bay and Strathfield) near Concord Hospital in Sydney, Australia, were recruited using a population-wide sampling frame of the compulsorily-registered New South Wales Electoral Roll. Men living in an aged care facility were excluded from the study, mainly due to feasibility reasons. Participants completed baseline assessments through self-completion questionnaires, interviewer-administered questionnaires, and clinical assessments. Baseline participation was 54% of those who were originally contacted. The primary reasons given for non-participation were lack of time or interest and health issues (Cumming et al., 2009).

Following the baseline phase in 2005–2007 (n = 1705), four additional phases have been conducted, with the first follow-up in 2007–2009 (n = 1366), the second in 2012–2013 (n = 954), the third in 2015–2016 (n = 735), and the most recent in 2019–2020 (n = 396) (Khalatbari-Soltani et al., 2022, Khalatbari-Soltani et al., 2021, Khalatbari-Soltani et al., 2021). Data linkage was also undertaken as part of the follow-up. In this study, we used data from baseline, linked to follow-up mortality data.

### 2.2 Exposures: Socioeconomic indicators and social mobility

We used three indicators of socioeconomic indicators, which were measured at the CHAMP baseline phase. A three-level ranking of highest qualification was used to assess educational attainment, including ‘high’ (university degree), ‘middle’ (trade, diploma, apprenticeship, or certificate), and ‘low’ (no post-school qualification), which correspond to categories used in the International Standard Classification of Education (*ISCED, 2011* *Operational Manual Guidelines for Classifying National Education Programmes and Related Qualifications*, 2015). The longest held occupation was first classified into eight major groups based on the Australian and New Zealand Standard Classification of Occupations (ANZSCO) (Trewin, Trewin & B, 2006), which are broadly similar to the International Standard Classification of Occupations (ISCO) (*Australian Bureau of Statistics (2022)*. ANZSCO is a skill-based classification that defines occupational groupings based on levels of education, knowledge, responsibility, training, and experience. These groupings are hierarchically ordered, with those occupations that have the highest skill requirements being at the top of the hierarchy. As per previous studies (Australian Bureau of Statistics., 2021; Turrell, Stanley, de Looper, & Oldenburg, 2006), using this skill-based hierarchical ordering, three main categories of occupational position were then defined: ‘high’ (higher professionals and managers, lower professionals and managers, and higher clerical services), ‘middle’ (small employers and self-employed, farmers, lower supervisors, and technicians), and ‘low’ (lower clerical, services, sales workers, skilled and unskilled workers). Participants reported their sources of income by selecting from the following six response options (more than one response allowed): ‘age pension’, ‘repatriation pension/veteran's pension’, ‘superannuation or other private income’, ‘own business/farm/partnership’, ‘wage or salary’, and ‘other (please specify)’. Source of income in older age was categorised based on Australia's retirement income system which comprises a means-tested age pension, mandatory superannuation, and voluntary savings (Chomik & Piggott, 2012). Following this, three categories were specified: ‘high’ (sources of income without government pension), ‘middle’ (government pension with other sources of income), and ‘low’ (only government pension).

We used these three socioeconomic indicators to describe the trajectories of intra-generational social mobility across three stages of the life-course, as has been done before (van de Straat et al., 2020). Educational level was used as a socioeconomic indicator for late adolescence to early adulthood as it was measured based on the highest qualification achieved, which commonly assesses educational experiences at these early life stages (Khalatbari-Soltani, Maccora, Blyth, Joannès, & Kelly-Irving, 2022). Occupational position was used as a mid-life socioeconomic indicator, as it was measured based on the longest-held occupation (i.e. during adult life). Sources of income were used as an older age socioeconomic indicator as we assessed participants’ sources of income when they were 70 years old or over. We, therefore, did not consider sources of income as a mid-life socioeconomic indicator given the nature of our measurement.

**Fig. 1:**
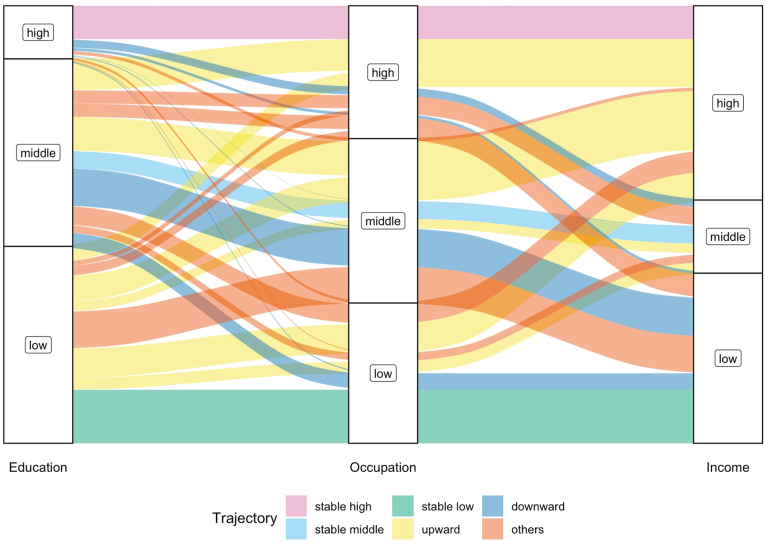
Movements between socioeconomic conditions across the life-course for different social mobility trajectories. Note: The widths of the lines are proportionate to the numbers of people in the corresponding trajectories. The height of the sections within each bar (low, middle, and high) represents the numbers of people in that category across the whole sample.

Having defined three levels of socioeconomic conditions (high, middle, and low) at each of the three life stages, a permutated total of 27 trajectories could be mapped to describe participants’ intra-generational social mobility (Fig. 1). These were used to test the falling from grace, rising from rags, and dissociative hypotheses.

To examine the falling from grace/rising from rags hypotheses, we constructed a social mobility trajectory variable with six categories: (1) stable high (never exposed to socioeconomic disadvantage); (2) stable middle; (3) stable low (always exposed to socioeconomic disadvantage); (4) upward mobility; (5) downward mobility; and (6) mixed. Interpretation must be tailored to reflect the fact that mobility and accumulation are inherently intertwined (Hallqvist, Lynch, Bartley, Lang, & Blane, 2004). The falling from grace/rising from rags hypotheses were considered to hold if only downward/upward trajectories had stronger associations with mortality when compared to stable high. Conversely, if being in the stable low category had a stronger association with increased mortality risk compared to being in the upward or downward trajectories, then the accumulation hypothesis was considered to hold (Carmeli et al., 2020; Howe et al., 2016). To disentangle impacts of social mobility from accumulation, we created a second social mobility trajectory variable with four categories which collapsed all non-mobile categories: (1) non-mobile (stable high, low, and middle); (2) upward mobility; (3) downward mobility; and (4) mixed. To assess the dissociative hypothesis (non-directional explanation), we created a social mobility status variable with two categories: (1) non-mobile (stable high, stable middle, or stable low); and (2) mobile (upward, downward, or any other forms of mobility). The classifications of these variables are detailed in Supplementary Table S1 and Table 1.

**Table 1:** Summary of social mobility variables and reference categories based on the hypotheses.

Social mobility variables and the terms used	Categories	Hypothesis tested	Reference category^a^
**Theory-driven social mobility trajectories**	**Six categories** Stable high Stable middle Stable low Upward Downward Mixed	Falling from grace	Stable high
		Rising from rags	Stable low

**Four categories** Non-mobile Upward Downward Mixed	Social mobility vs accumulation	Non-mobile

**Theory-driven social mobility status**	Mobile Non-mobile	Dissociative hypothesis	Non-mobile

**Data-driven social mobility trajectories**	Stable high Stable low Mixed	Impact of data-driven trajectories on mortality	Stable high

a Detailed definitions of categories are provided in Supplementary Table S1.

### 2.3 Outcome: all-cause mortality

The outcome was all-cause mortality, obtained from the New South Wales Registry of Births, Deaths and Marriages (RBDM; state government records of all deaths in New South Wales; https://www.nsw.gov.au/births-deaths-marriages), and linked to CHAMP data via the Centre for Health Record Linkage (http://www.cherel.org.au/) using probabilistic record linkage methods and Choice-Maker software. Mortality follow-up was available from January 1, 2005 to December 31, 2017.

### 2.4 Covariates

The following covariates were included in this study as potential confounders: age (continuous), living arrangement (living with others/living alone), and country of birth (Australian-born/other). To avoid overadjustment bias (van Zwieten et al., 2022), a priori, we decided not to adjust for factors such as comorbidities and health-related behaviours (Khalatbari-Soltani et al., 2022, Khalatbari-Soltani et al., 2021, Khalatbari-Soltani et al., 2021; Khalatbari-Soltani et al., 2020), as they lie on the causal pathway between socioeconomic conditions and mortality.

### 2.5 Statistical analyses

Statistical analyses were performed using R (version 4.1.2, R Foundation for Statistical Computing, Vienna, Austria). Individuals with missing exposure, covariate, or outcome data were excluded from the analysis. For descriptive analysis of the cohort, we calculated mean (standard deviation) and N (%) as appropriate to the variable. For comparing characteristics across categories, we used Chi-square tests for categorical variables and Mann–Whitney tests for continuous variables.

#### 2.5.1 Associations of social mobility status and trajectories with mortality

Survival time was calculated from the date of baseline interview to the date of death or censoring at the end of follow-up (December 31, 2017). Unadjusted mortality rates per 1000 person-years were calculated by social mobility trajectory and status. We also calculated the age and country of birth adjusted mortality rates per 1000 person-years using Poisson regression— a commonly used method when the outcome is not rare (Greenland, 2004). Survival curves were estimated using unadjusted Kaplan-Meier curves. For the theory-based analyses, we examined the associations of social mobility trajectory (reference category: stable high) and social mobility status (reference category: non-mobile) with mortality by using univariable and multivariable Cox proportional hazards regression models (‘survival’ package in R) (Therneau T.M., 2021). Survival time was measured as the time from the date of baseline interview (January 1, 2005) to either the date of death or end of follow-up (December 31, 2017). Multivariable models were adjusted for age, country of birth, and living arrangement. Unadjusted and adjusted hazard ratios (HR) and 95% confidence intervals (CI) were reported. The proportional hazards assumption was assessed using Schoenfeld residuals; in all models, this assumption was satisfied. Post-hoc, we reran the social mobility trajectory analyses with stable low as the reference category (instead of stable high), as this reference group was considered more appropriate for testing the rising from rags hypothesis. We also conducted an exploratory analysis where we compared upward, downward, and mixed social mobility trajectories to a single non-mobile category that collapsed all stable categories. This was for comparability to previous studies and to enable examination of the social mobility hypotheses without the social gradient and accumulation effects present in the main analysis. A summary of all social mobility variables and analyses is provided in Table 1. A priori, we also examined whether the associations between social mobility trajectory and status and mortality varied by country of birth. This was because CHAMP is an ethnically diverse cohort with half of the sample born overseas and previous interactions were found between country of birth and socioeconomic conditions with other health outcomes (Khalatbari-Soltani, Stanaway, et al., 2021).

#### 2.5.2 Data-driven classification of social mobility trajectories and mortality

We used the poLCA package in R to conduct a Latent Class Analysis (LCA) (Linzer & Lewis, 2011) to identify data-driven social mobility trajectories that we could compare to our previous approaches. LCA clusters categories of individuals who share similar combinations of socioeconomic indicators across the life-course into distinct classes. We fitted models with two to six classes. The optimal number of classifications was identified based on the classes being theoretically meaningful and by using Akaike Information Criterion (AIC), Bayesian Information Criterion (BIC), and scaled entropy (ranging from zero to one), with the lowest values of AIC or BIC or the closest-to-one entropy indicating the best data-driven model. We then used a Cox proportional hazards model to explore the association between the latent classes of social mobility and mortality, with adjustment for the same potential confounders as the theory-driven models.

## 3 Results

### 3.1 Participant characteristics

Of 1705 participants at baseline, 137 were excluded due to refusal of mortality data linkage (n = 66), missing socioeconomic indicator data (n = 49), or missing covariates (n = 22), leaving a total of 1568 (92%) men for analysis (Supplementary Fig. S1). Excluded participants were more likely to be overseas-born but did not differ in terms of age or living arrangements (Supplementary Table S2).

Table 2 summarises the baseline characteristics of the participants by theory-driven social mobility trajectory and status. The mean age of study participants at baseline was 76.8 (SD 5.4) and most (72.0%) were less than 80 years old. The proportion of Australian-born men (50.7%) was similar to that of overseas-born men (49.3%) and less than 20% of participants lived alone. Considering social mobility trajectories, moving upward was the predominant trajectory (36.0%), followed by mixed trajectories (25.1%), then downward (15.1%), stable low (12.2%), stable high (7.6%), and finally stable middle (4.0%). Over three-fourths of the participants were classified as having mobile social mobility status (76.2%), while the remainder were non-mobile (23.8%). All specific social mobility trajectories are visualised in Fig. 1.

**Table 2:** Baseline characteristics of participants overall and by social mobility trajectory and social mobility status.

Characteristics	Overall (n = 1568)	Social mobility trajectory						Social mobility status	
		Stable high (n = 119)	Stable middle (n = 62)	Stable low (n = 192)	Upward (n = 565)	Downward (n = 236)	Mixed (n = 394)	Non-mobile (n = 373)	Mobile (n = 1195)
Age (years), mean ± SD	76.8 ± 5.4	76.5 ± 5.0	76.1 ± 5.8	76.5 ± 4.8	77.1 ± 5.7	76.5 ± 5.0	76.8 ± 5.4	76.4 ± 5.0	76.9 ± 5.5
Age categories, n (%)									
70–79	1132 (72.2)	85 (71.4)	51 (82.3)	149 (77.6)	385 (68.1)	177 (75.0)	285 (72.3)	285 (76.4)	847 (70.9)
≥80	436 (27.8)	34 (28.6)	11 (17.7)	43 (22.4)	180 (31.9)	59 (25.0)	109 (27.7)	88 (23.6)	348 (29.1)
Country of birth, n (%)									
Australian-born	795 (50.7)	89 (74.8)	41 (66.1)	41 (21.4)	345 (61.1)	103 (43.6)	176 (44.7)	171 (45.8)	624 (52.2)
Other	773 (49.3)	30 (25.2)	21 (33.9)	151 (78.6)	220 (38.9)	133 (56.4)	218 (55.3)	202 (54.2)	571 (47.8)
Living alone, n (%)	289 (18.4)	19 (16.0)	16 (25.8)	24 (12.5)	101 (17.9)	52 (22.0)	77 (19.5)	230 (19.2)	59 (15.8)

SD: standard deviation.

Across the categories of social mobility trajectory or status, there were no important differences in the average age of the participants (Table 2). Australian-born men were more likely to have experienced stable high, stable middle, and upward social mobility trajectories than overseas-born men, while overseas-born men accounted for higher proportions of the stable low and downward trajectories. Considering the social mobility trajectories, the highest proportion living alone was observed among men with stable middle (25.8%) and downward trajectories (22.0%), while the proportion living alone between mobile and non-mobile men did not differ significantly (19.2% vs. 15.8%).

During a mean follow-up time of 9.1 (SD 3.6) years, 797 deaths occurred (Table 3). Regarding social mobility trajectories and mortality rates, men in the stable low category had the highest mortality rate, followed by the downward category, then stable middle, mixed, and upward, with the stable high category having the lowest mortality rate. Mortality rates were similar between the mobile and non-mobile categories (Table 3).

**Table 3:** Mortality rates by social mobility status and social mobility trajectory.

	n	Mortality			
		Person-years^a^	Deaths	Unadjusted rate^b^ (95% CI)	Adjusted rate^b^^,^^c^ (95% CI)
**Overall**	1568	14,198	797	56.1 (52.3–60.2)	53.3 (50.6–56.3)
**Social mobility trajectory**					
Stable high	119	1169	50	42.8 (31.7–56.4)	38.7 (30.4–49.3)
Stable middle	62	547	33	60.3 (41.5–84.7)	64.1 (47.8–86.1)
Stable low	192	1659	105	63.3 (51.8–76.6)	73.1 (61.4–87.1)
Upward	565	5153	273	53.0 (46.9–59.7)	50.1 (45.3–55.3)
Downward	236	2103	130	61.8 (51.7–73.4)	65.5 (56.4–76.3)
Mixed	394	3567	206	57.8 (50.1–66.2)	58.1 (51.8–65.2)
**Social mobility status**					
Non-mobile	373	3375	188	55.7 (48.0–64.3)	58.0 (51.3–65.5)
Mobile	1195	10,823	609	56.3 (51.9–60.9)	55.6 (52.4–59.0)

Note: Table 1 defines social mobility variables and reference categories based on the hypotheses.

a We used calendar year as the time scale and measured as the time from the date of baseline interview (January 1, 2005) to either the date of death or end of follow-up (December 31, 2017).

b The rates are per 1000 person-years.

c The rates are adjusted for age and country of birth using Poisson regression.

### 3.2 Associations of social mobility status and trajectories with mortality

**Fig. 2:**
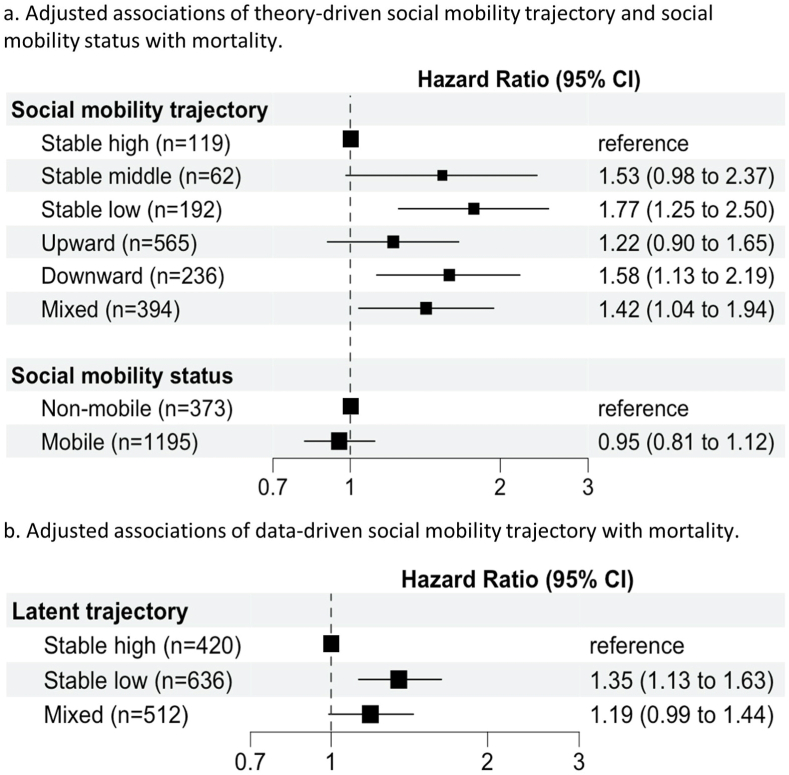
Adjusted associations of theory-based social mobility trajectory, theory-based social mobility status, and data-driven social mobility trajectory with mortality. Note: n = 1568; Hazard ratios are from multivariable Cox proportional-hazards models, adjusted for age, country of birth, and living arrangement. CI: confidence interval. HR: hazard ratio.

Fig. 2 depicts the adjusted associations of social mobility trajectory and status with mortality. Regarding social mobility trajectories, when compared to the stable high reference category, a stable low social mobility trajectory was associated with a 77% higher risk of mortality (95% CI: 1.25 to 2.50). Men with downward social mobility trajectories had 58% increased risks of mortality (95% CI: 1.13 to 2.19) compared to those with stable high trajectories. A similar increase in mortality risk was found among those in the stable middle trajectory, although this was not significant (HR = 1.53; 95% CI: 0.98 to 2.37). Those with mixed trajectories also had an increased mortality risk of 42% compared to those with stable high trajectories (HR = 1.42, 95% CI: 1.04–1.94). Meanwhile, mortality risk for those with upward social mobility trajectories was not statistically different from those with stable high trajectories (HR = 1.22, 95% CI: 0.90 to 1.65) (Fig. 2a). In our exploratory analysis that considered those with stable low trajectories as the reference group, those with upward social mobility trajectories had a 31% lower mortality risk (HR = 0.69, 95% CI 0.55 to 0.88); while those with stable high had the lowest risk of mortality (HR = 0.57, 95% CI 0.40 to 0.80) (Supplementary Fig. S2a). In our exploratory analysis considering all non-mobile trajectories combined as the reference category, the mortality risk of those with either upward (HR = 0.86, 95% CI: 0.71 to 1.04) or downward (HR = 1.10, 95% CI: 0.88 to 1.37) social mobility trajectories was not statistically significantly different from those with non-mobile trajectories (Supplementary Fig. S2b).

Considering the dissociative hypothesis, no association was evident between mobile social mobility status and mortality (HR = 0.95, 95% CI: 0.81 to 1.12) compared to non-mobile status (Fig. 2a). Unadjusted associations of social mobility trajectories and status with mortality are shown in Supplementary Figs. S3 and S4; unadjusted results were similar to the adjusted results. No statistical interactions were found between country of birth and social mobility status or trajectory (all p_interaction_ > 0.05).

### 3.3 Data-driven classification of social mobility trajectories and mortality

Model fit statistics of the data-driven classification of social mobility trajectories are presented in Supplementary Table S3. After comparing the AIC, BIC, and entropy values of all the LCA models and considering whether classes were theoretically meaningful, the classification with three classes was determined to be the best data-driven model. The distribution of the socioeconomic indicators across life stages for these three LCA-derived classes is shown in Supplementary Fig. S5. Class 1 was mostly characterised by high socioeconomic conditions, being denoted as ‘stable high’ (n = 420, 26.8%). Class 2 was mostly characterised by low socioeconomic conditions, being denoted as ‘stable low’ (n = 636, 40.6%). Class 3 had variable socioeconomic condition levels across the life-course (socioeconomic conditions typically remained at the middle level during the first two stages before splitting into low and high levels at the last stage), being denoted as ‘mixed’ (n = 512, 32.6%).

Results of the Cox regression model using the data-driven social mobility trajectories are shown in Fig. 2b. Compared to the stable high reference category, men with data-driven stable low trajectories had a higher mortality risk (HR = 1.35, 95% CI: 1.13 to 1.63) and men in the mixed trajectories also appeared to have slightly increased risks of mortality although this result was not statistically significant (HR = 1.19, 95% CI: 0.99 to 1.44).

## 4 Discussion

To the best of our knowledge, our study is one of the first to investigate the life-course association between intra-generational social mobility and mortality in older age with a combination of theory-based hypothesis approaches and a data-driven approach, informed by three hypotheses (falling from grace, rising from rags, and dissociative hypotheses). With regard to the falling from grace hypothesis, men experiencing downward social mobility trajectories had a higher risk of mortality compared to those with stable high social mobility trajectories. However, those with the stable low social mobility trajectories had the greatest risk of mortality. This suggests that it may not be downward mobility per se that resulted in the increased mortality risks for this category, but rather the impacts of an intermediate duration of disadvantage, with the dominant process being one of accumulation in the effects of adverse socioeconomic conditions over time. Indeed, we found no support for the falling from grace and rising from rags hypotheses when we compared upward and downward social mobility trajectories to those with non-mobile trajectories in our exploratory analysis. For the rising from rags hypothesis, the upward category had lower mortality risk than the stable low category in our exploratory analysis, however, the lowest risk was among the stable high category. This again suggests that cumulative effects may be a key driver. Of note, our findings did not support the dissociative hypothesis, with men in the mobile category having similar mortality risks to those in the non-mobile category. The data-driven classification of social mobility from the exploratory analyses identified three classes of trajectories, including stable high, stable low, and mixed. In these analyses, the stable low social mobility trajectory had an increased risk of mortality compared to the stable high social mobility trajectory, while the mixed category had an intermediate mortality risk, again potentially supporting a cumulative effect of disadvantaged socioeconomic conditions across the life-course on mortality.

When we considered the stable high social mobility trajectory as our reference category, we mainly found a dose-response effect whereby a longer time exposed to disadvantaged socioeconomic conditions was associated with a higher risk of mortality. Specifically, we found that men with stable low life-course socioeconomic conditions had the highest risk of mortality compared with their stable high counterparts, which agrees with previous studies (Ericsson et al., 2019; Hart et al., 1998; Pensola & Martikainen, 2003). Men with downward social mobility trajectories also had a higher risk of mortality compared to those in the stable high category, which is in line with previous studies among men (Billingsley, 2012, 2019, 2020; Ericsson et al., 2019; Hart et al., 1998; Uggla & Billingsley, 2018), and men and women (Billingsley, 2020; Ericsson et al., 2019; Uggla & Billingsley, 2018). However, given that the risk of mortality for the downward category was not as high as those in the stable low category and was similar to that of the stable middle category, our findings indicate a predominance of the cumulative effect of adverse socioeconomic conditions rather than a mobility effect. We observed similar patterns when we considered the stable low social mobility trajectory as the reference category. As we were unable to disentangle cumulative effects from mobility effects (Hallqvist et al., 2004), we conducted an exploratory analysis seeking to address this issue by considering all immobile trajectories as the reference category, in which we also found no support for the social mobility hypotheses of falling from grace and rising from rags. Overall, our results support the cumulative effect model, highlighting the adverse impacts of cumulative exposure to disadvantaged socioeconomic conditions on health and well-being across the life-course, which in turn impacts mortality risk. Our findings may also reflect the importance of destination socioeconomic conditions, represented in the acculturation theory (Schumann et al., 2020).

We found no evidence to support the dissociative theory. This is in accordance with previous studies from different countries with a range of outcomes that have not found strong directionless social mobility effects (Jonsson et al., 2017; McLoughlin et al., 2023; Mohd Khairuddin, Bernabé, & Delgado-Angulo, 2021). We note that in proposing this hypothesis, Sorokin particularly emphasised the negative consequences of upward trajectories for mental health outcomes (Sorokin, 1927). It is possible that these processes may not equally apply to other outcomes such as mortality. Additionally, one study highlighted that individual perceptions of one's own social mobility might be different from objective measurements of socioeconomic mobility; which might be a potential explanation for the lack of social mobility status effect on mortality in our study (Präg & Gugushvili, 2021). This links to the growing body of literature on the importance of perceived social status and its associations with health outcomes (Cundiff & Matthews, 2017).

The trajectories (stable high, stable low, and mixed) identified through data-driven approaches differed from the theory-based hypothesis. While LCA identifies a certain number of discrete latent trajectories based on the data (Hossin et al., 2022), there is no proof that such discrete trajectories actually exist and remain as observed over time. Thus, applying both theory-driven and data-driven approaches to develop mobility trajectories is important for thorough examination of life-course social mobility trajectories (Warren, Luo, Halpern-Manners, Raymo, & Palloni, 2015). The data-driven stable low social mobility trajectory was associated with a higher mortality risk relative to those in the stable high category, consistent with the findings from the theory-based hypothesis and in agreement with previous literature (Hossin et al., 2022). Noticeably, men in the mixed category also seemed to have an increased mortality risk compared to those in the stable high category. This may again reflect cumulative effects such that men in this category had an intermediate duration of exposure to poor socioeconomic conditions and thus had an intermediate mortality risk between that of the stable high and stable low categories (which had the shortest and longest durations of exposure to poor socioeconomic conditions respectively).

Strengths of our study include the use of three different socioeconomic indicators in three distinct life-course periods to examine social mobility. Our study also had a precise measurement of mortality status with linked government data using probabilistic record linkage methods, and our models were adjusted for relevant confounders. A further strength is that the sociodemographic and health-related characteristics of the men in CHAMP are similar to the general population of older men in Australia (Holden et al., 2005). However, there are also some limitations of our study. Our analysis was limited to all-cause mortality; further studies are needed to examine cause-specific mortality outcomes. This may help to further elucidate the mechanisms through which socioeconomic conditions affect health. We recognise that similar to many previous studies on social mobility, we were unable to statistically separate the observed effects of social mobility from those of origin and destination (Bridger & Daly, 2020; Salmela et al., 2021) due to the limitations of available methods (Hallqvist et al., 2004; van der Waal, Daenekindt, & de Koster, 2017). While diagonal reference modeling (DRM) could help with this issue (Zang, Sobel, & Luo, 2023); we did not use DRM as it requires large sample sizes to be able to reliably detect mobility effects (Präg, Fritsch, & Richards, 2022) and simulation research has demonstrated that DRM may generate results that force mobility effects to zero (Ethan Fosse; Fabian T. Pfeffer, 2019). Given the limitations of available methods, differentiating empirically between these theoretical models is difficult. Nevertheless, previous research demonstrates that the distinction between these theoretical models is more conceptual than empirical, and the interconnectedness of these models needs to be considered (Hallqvist et al., 2004; Pudrovska & Anikputa, 2014). CHAMP only included men 70 years or older, which limits the generalisability of our results to people younger than 70 years, women, and gender-diverse people. Potential impacts of cultural differences, cohort differences, time- and context-specific socioeconomic characteristics of individuals also make generalisability of findings more challenging. Not including individuals living in a residential aged care facility at baseline in CHAMP, who are among the most frail members of society, may potentially have led to selection bias and associated underestimation of social mobility and mortality associations. Similarly, selection bias due to survival bias effects may also be present as CHAMP only included men aged 70 years and over; and men who die earlier may differ in terms of their social mobility compared to men who survive beyond this age. In addition, in the three local government areas (Burwood, Canada Bay, and Strathfield) chosen to participate in CHAMP, only 2% of households are ranked in the three lowest deciles of the Australia-wide Index of Relative Socioeconomic Disadvantage. The relative affluence of these suburbs points may have led to selection bias. Future studies are required to examine the generalisability of our findings across different populations. Socioeconomic indicators in early and mid-life were collected retrospectively and thus may be subject to recall bias, leading to potential misclassification. Nevertheless, recall of simple socioeconomic conditions among older adults is generally considered to yield reliable data (Lacey, Belcher, & Croft, 2012). Of note, we did not have repeated measures of occupational position and sources of income, and thus, we were unable to account for potential changes in these socioeconomic exposures across the life course. We also did not have a measure of parental socioeconomic conditions to enable examination of inter-generational dynamics and acculturation processes (Blau, 1956).

## 5 Conclusion

In a representative sample of Australian men aged ≥70 years old, our findings provide support for a predominant role of cumulative effects of disadvantaged socioeconomic conditions over effects of social mobility. The dose-response relationships between adverse socioeconomic experiences over time and mortality highlight the need for targeted interventions to address adverse socioeconomic conditions across the life-course.

## Ethics approval

The CHAMP study was approved by a local Health Service Human Research Ethics Committee. Written informed consent was obtained from all participants.

## Author statement

SKS conceived and designed the study. AvZ and KK helped with study design. SKS and MHT had full access to data and take responsibility for the integrity and accuracy of the data. MHT performed the statistical analyses and wrote the first draft with help from SKS, AvZ, and KK. Interpretation of the data: MHT, SKS, AvZ, KK, FMB, DGLC, DJH, VN, MJS, LMW, and RGC; Substantively and critically revised the manuscript for important intellectual content: SKS, AvZ, KK, FMB, DGLC, DJH, VN, MJS, LMW, and RGC. All authors read and approved the final version of the manuscript.

## Funding and role of the funding source

SKS is supported by the Australian Research Council Centre of Excellence in Population Ageing Research (Project number CE170100005). The funder had no role in preparation of the manuscript and decision to publish.

## Declaration of competing interest

The authors report no conflict of interest.

## Data Availability

Some access restrictions apply to the data underlying this study’s findings. The original human ethics committee approval for the Concord Health and Ageing in Men Project (CHAMP) in 2004 did not allow for data to be sent outside Australia. Furthermore, the participants in CHAMP have not consented to their data being distributed beyond the CHAMP investigators and their associates. Qualified researchers may submit a request to the CHAMP Management Committee (vasi.naganathan@sydney.edu.au) and access will require additional ethics approval from the Sydney LHD HREC - CRGH, including considerations of privacy for data sharing.
