# Supplementary material for: Intra-generational social mobility and mortality among older men in the Concord Health and Ageing in Men Project: A cohort study
Source: SSM Popul Health. 2023 Dec 11;25:101581. doi: 10.1016/j.ssmph.2023.101581 (PMC10803938; doi:10.1016/j.ssmph.2023.101581)
Supplement: Multimedia component 1 [file mmc1.docx]

**SUPPLEMENTARY INFORMATION**

**Checklist 1.** STROBE Statement—Checklist of items that should be included in reports of *cohort studies*.

|  | Item No | Recommendation |
| --- | --- | --- |
| **Title and abstract** | 1 | (*a*) Indicate the study’s design with a commonly used term in the title or the abstract |
|  |  | (*b*) Provide in the abstract an informative and balanced summary of what was done and what was found |
| Introduction | | |
| Background/rationale | 2 | Explain the scientific background and rationale for the investigation being reported  **Introduction (paragraphs 1, 2, and 3)** |
| Objectives | 3 | State specific objectives, including any prespecified hypotheses  **Introduction (paragraph 4)** |
| Methods | | |
| Study design | 4 | Present key elements of study design early in the paper  **Methods (paragraph 1)** |
| Setting | 5 | Describe the setting, locations, and relevant dates, including periods of recruitment, exposure, follow-up, and data collection  **Methods (paragraph 1)** |
| Participants | 6 | (*a*) Give the eligibility criteria, and the sources and methods of selection of participants. Describe methods of follow-up  **Methods (paragraph 1), Figure S1** |
|  |  | (*b*) For matched studies, give matching criteria and number of exposed and unexposed  **N/A** |
| Variables | 7 | Clearly define all outcomes, exposures, predictors, potential confounders, and effect modifiers. Give diagnostic criteria, if applicable  **Methods (Page 6-8)** |
| Data sources/ measurement | 8* | For each variable of interest, give sources of data and details of methods of assessment (measurement). Describe comparability of assessment methods if there is more than one group  **Methods (Page 6-8)** |
| Bias | 9 | Describe any efforts to address potential sources of bias  **Methods (Page 8-9, Statistical Analysis)** |
| Study size | 10 | Explain how the study size was arrived at  **Figure S1** |
| Quantitative variables | 11 | Explain how quantitative variables were handled in the analyses. If applicable, describe which groupings were chosen and why  **Methods (Page 6–8)** |
| Statistical methods | 12 | (*a*) Describe all statistical methods, including those used to control for confounding  **Methods (Statistical Analysis)** |
|  |  | (*b*) Describe any methods used to examine subgroups and interactions  **Methods (Statistical Analysis)** |
|  |  | (*c*) Explain how missing data were addressed  **Methods (Statistical Analysis)** |
|  |  | (*d*) If applicable, explain how loss to follow-up was addressed  **Methods (Statistical Analysis)** |
|  |  | (*e*) Describe any sensitivity analyses  **Methods (Statistical Analysis)** |
| Results | | |
| Participants | 13* | (a) Report numbers of individuals at each stage of study—eg numbers potentially eligible, examined for eligibility, confirmed eligible, included in the study, completing follow-up, and analysed  **Results (paragraph 1), Figure S1, Table S2** |
|  |  | (b) Give reasons for non-participation at each stage  **Methods (paragraph 1), Figure S1, Table S2- we also referred to a previous study that described reasons for non-participation at each follow-up time of the CHAMP study.** |
|  |  | (c) Consider use of a flow diagram  **Figure S1** |
| Descriptive data | 14* | (a) Give characteristics of study participants (eg demographic, clinical, social) and information on exposures and potential confounders  **Table 1, Table S2** |
|  |  | (b) Indicate number of participants with missing data for each variable of interest  **Table S2, Figure S1** |
|  |  | (c) Summarise follow-up time (eg, average and total amount)  **Table 2** |
| Outcome data | 15* | Report numbers of outcome events or summary measures over time  **Table 2** |
| Main results | 16 | (*a*) Give unadjusted estimates and, if applicable, confounder-adjusted estimates and their precision (eg, 95% confidence interval). Make clear which confounders were adjusted for and why they were included  **Results (Page 10-12), Figure S3, Figure S4** |
|  |  | (*b*) Report category boundaries when continuous variables were categorized  **Table 1-2, and Supplementary Tables** |
|  |  | (*c*) If relevant, consider translating estimates of relative risk into absolute risk for a meaningful time period  **Table 2** |
| Other analyses | 17 | Report other analyses done—eg analyses of subgroups and interactions, and sensitivity analyses  **Tables S3, Figure S5** |
| Discussion | | |
| Key results | 18 | Summarise key results with reference to study objectives  **Discussion (paragraph 1)** |
| Limitations | 19 | Discuss limitations of the study, taking into account sources of potential bias or imprecision. Discuss both direction and magnitude of any potential bias  **Discussion (paragraph 7)** |
| Interpretation | 20 | Give a cautious overall interpretation of results considering objectives, limitations, multiplicity of analyses, results from similar studies, and other relevant evidence  **Discussion** |
| Generalisability | 21 | Discuss the generalisability (external validity) of the study results  **Discussion** |
| Other information | | |
| Funding | 22 | Give the source of funding and the role of the funders for the present study and, if applicable, for the original study on which the present article is based |

*Give information separately for exposed and unexposed groups.

**Note:** An Explanation and Elaboration article discusses each checklist item and gives methodological background and published examples of transparent reporting. The STROBE checklist is best used in conjunction with this article (freely available on the Web sites of PLoS Medicine at http://www.plosmedicine.org/, Annals of Internal Medicine at http://www.annals.org/, and Epidemiology at http://www.epidem.com/). Information on the STROBE Initiative is available at http://www.strobe-statement.org.

**Supplementary Figure S1.** Sample selection flowchart

Overall baseline sample (n=1705)

Total sample for analysis (n=1568, 92.0%)

Refusal of mortality data linkage (n=66, 3.9%)

Missing social mobility data (n=49, 2.9%)

Missing covariates (n=22, 1.3%)

**Supplementary Figure 2.** Exploratory analyses for the association between social mobility trajectory and mortality.


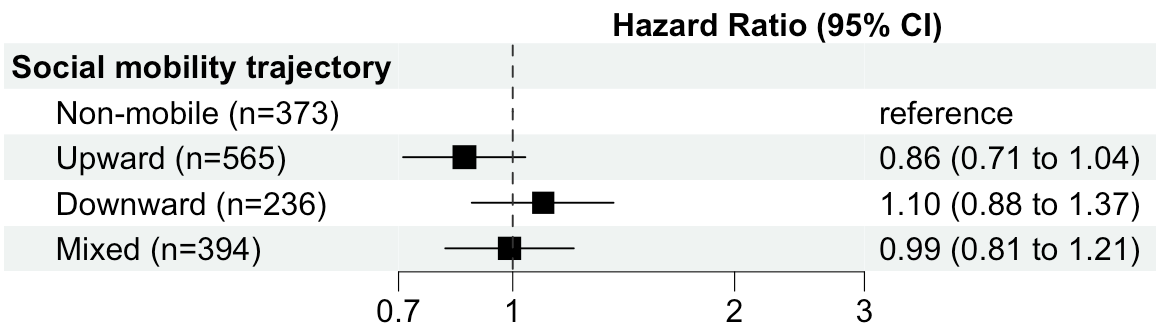


b. Exploratory analysis - adjusted associations between social mobility trajectory variable with four categories and mortality

a. Exploratory analysis - adjusted associations between social mobility trajectory and mortality – stable low as the reference group


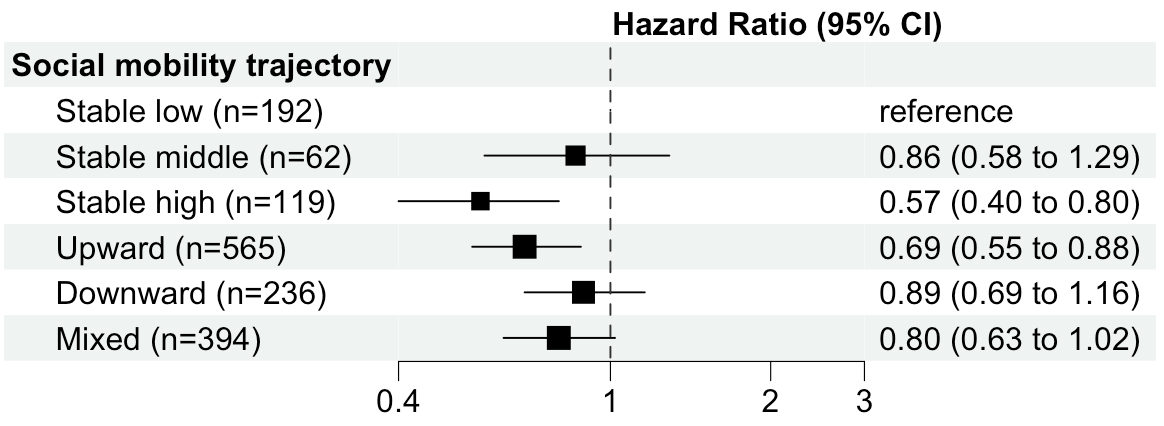


*Note:* n=1568; Hazard ratios are from multivariable Cox proportional-hazards models, adjusted for age, country of birth, and living arrangement. CI: confidence interval. HR: hazard ratio.

Note: Table 1 defines social mobility variables and reference categories based on the hypotheses.

**Supplementary Figure S3.** Kaplan–Meier curves of (a) social mobility trajectory and (b) social mobility status with mortality.


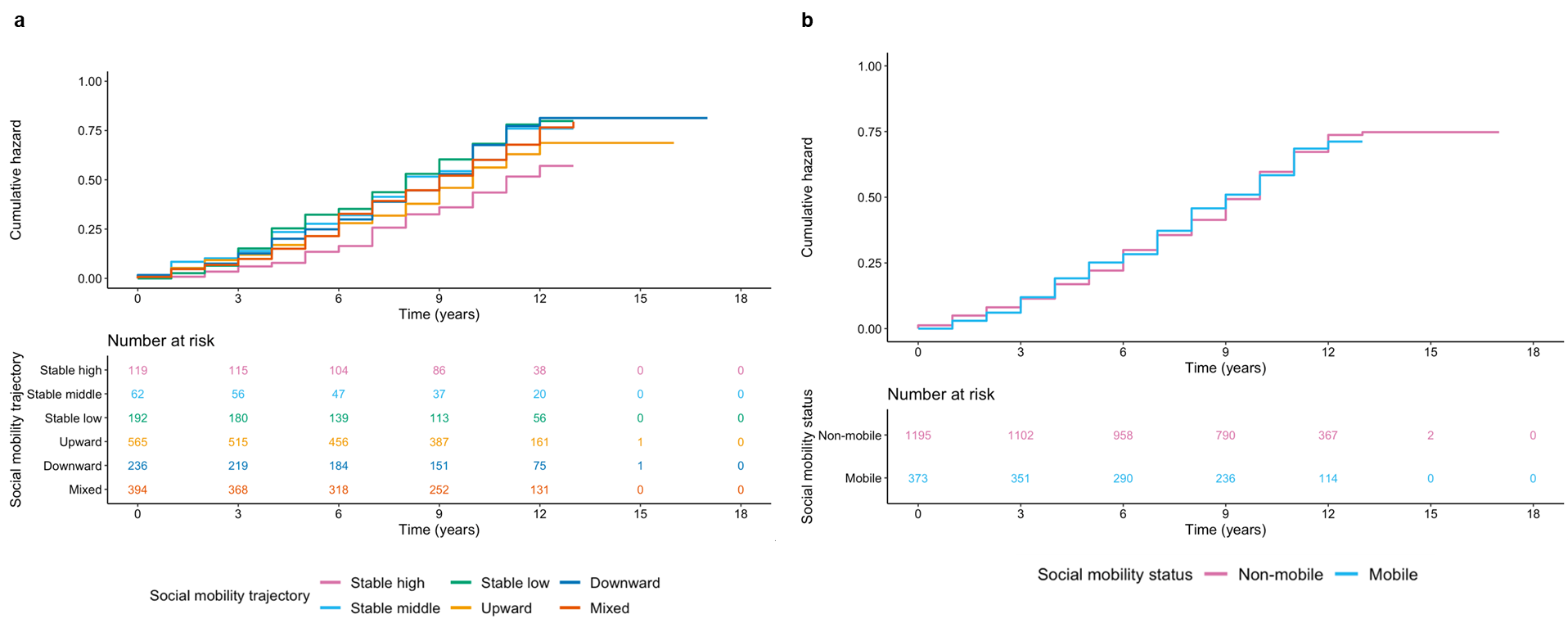


**Supplementary Figure S4.** Unadjusted associations of social mobility trajectory and social mobility status with mortality.


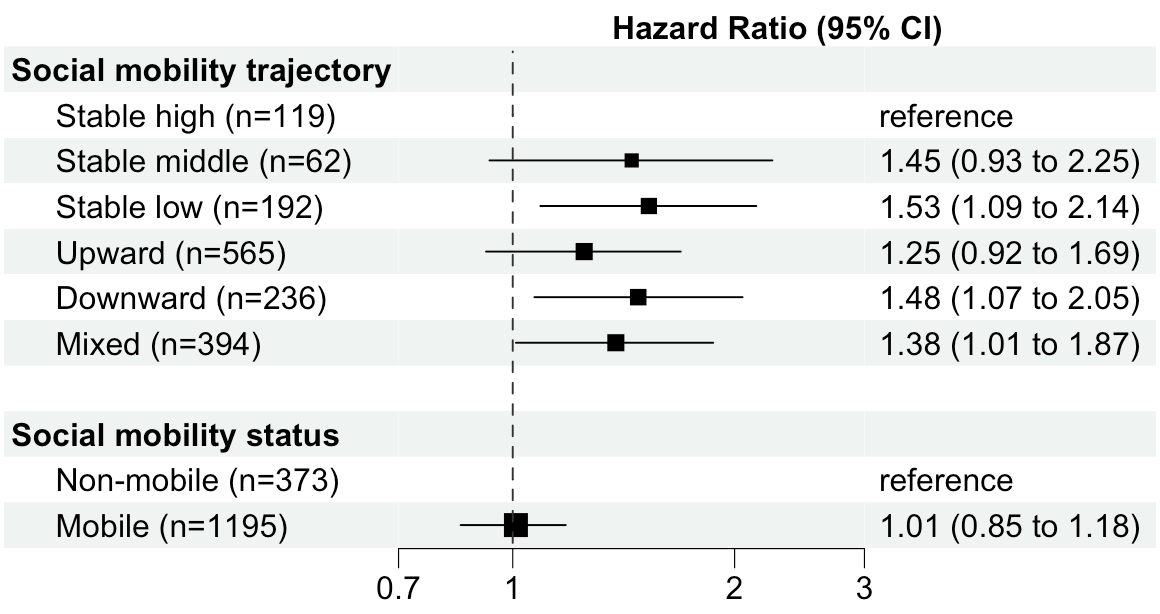


*Note:* n=1568; Hazard ratios are from multivariable Cox proportional-hazards models, adjusted for age, country of birth, and living arrangement. CI: confidence interval. HR: hazard ratio.

Note: Table 1 defines social mobility variables and reference categories based on the hypotheses.

**Supplementary Figure S5.** Distribution of the socioeconomic indicators across the three optimal classes from latent class analysis (LCA).
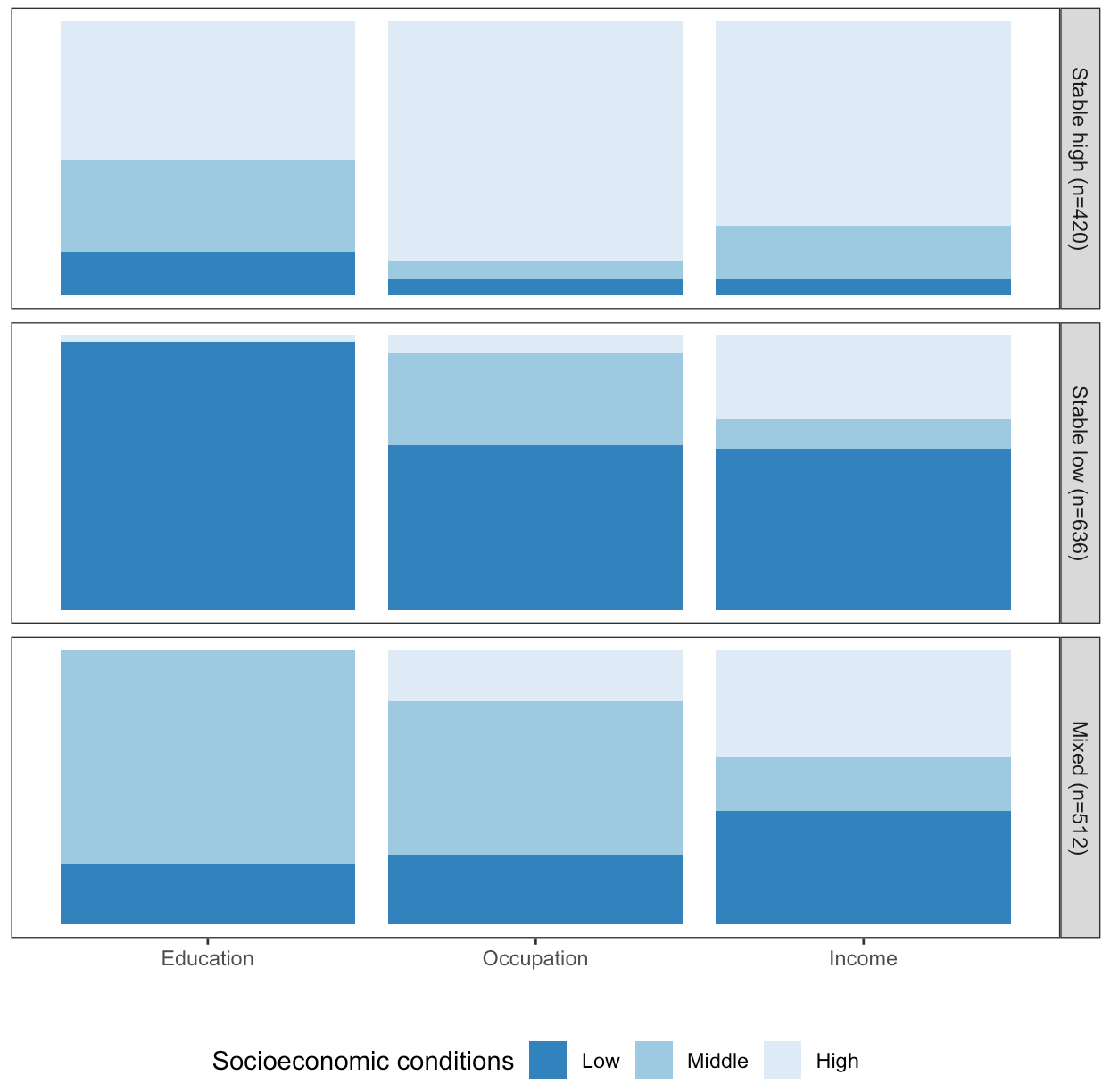


**Supplementary Table S1.** Classification of social mobility trajectory and social mobility status.

| **Variable** | **Categories included, defined by socioeconomic conditions at each life stage** |
| --- | --- |
| **Social mobility trajectory with six categories** | |
| Stable high | High-high-high |
| Stable middle | Middle-middle-middle |
| Stable low | Low-low-low |
| Upward | Low-low-middle, low-low-high, low-middle-middle, low-middle-high, low-high-high, middle-middle-high, middle-high-high |
| Downward | High-high-middle, high-high-low, high-middle-middle, high-middle-low, high-low-low, middle-middle-low, middle-low-low |
| Mixed | All others (high-middle-high, high-low-high, high-low-middle, middle-high-middle, middle-high-low, middle-low-middle, middle-low-high, low-high-low, low-high-middle, low-middle-low) |
| **Social mobility trajectory with four categories** | |
| Non-mobile | High-high-high, middle-middle-middle, low-low-low |
| Upward | Low-low-middle, low-low-high, low-middle-middle, low-middle-high, low-high-high, middle-middle-high, middle-high-high |
| Downward | High-high-middle, high-high-low, high-middle-middle, high-middle-low, high-low-low, middle-middle-low, middle-low-low |
| Mixed | All others (high-middle-high, high-low-high, high-low-middle, middle-high-middle, middle-high-low, middle-low-middle, middle-low-high, low-high-low, low-high-middle, low-middle-low) |
| **Social mobility status** |  |
| Non-mobile | High-high-high, middle-middle-middle, low-low-low |
| Mobile | All others |

**Supplementary Table S2.** Characteristics of participants included and excluded from the analyses.

| **Characteristic** | **Included** | **Excluded** | **P-value^a^** |
| --- | --- | --- | --- |
|  | **(n=1568)** | **(n=137)** |  |
| Age, *years* | 76 (72–80) | 75 (72–80) | 0.44 |
| Age categories, % |  |  | 0.98 |
| 70–79 | 72.2 | 72.1 |  |
| ≥ 80 | 27.8 | 27.9 |  |
| Country of birth, % |  |  | 0.01 |
| Australian-born | 50.7 | 39.4 |  |
| Other | 49.3 | 60.6 |  |
| Living alone, % | 18.4 | 21.2 | 0.16 |

^a^P-values calculated using Chi-square test for categorical variables and Mann–Whitney test for continuous variables.

**Supplementary Table S3.** Goodness-of-fit indices of latent class models with 2 to 6 classes.

| **Number of classes** | **AIC** | **BIC** | **Entropy** |
| --- | --- | --- | --- |
| 2 | 9269.663 | 9339.311 | 0.710 |
| 3 | 9192.571 | 9299.723 | 0.637 |
| 4 | 9202.912 | 9347.566 | 0.634 |
| 5 | 9215.711 | 9397.868 | 0.479 |
| 6 | 9228.996 | 9448.656 | 0.432 |
| AIC: Akaike Information Criterion; BIC: Bayesian Information Criterion | | | |
